# A FERONIA-Like Receptor Kinase Regulates Strawberry (*Fragaria* × *ananassa*) Fruit Ripening and Quality Formation

**DOI:** 10.3389/fpls.2017.01099

**Published:** 2017-06-28

**Authors:** Meiru Jia, Ning Ding, Qing Zhang, Sinian Xing, Lingzhi Wei, Yaoyao Zhao, Ping Du, Wenwen Mao, Jizheng Li, Bingbing Li, Wensuo Jia

**Affiliations:** College of Horticulture, China Agricultural UniversityBeijing, China

**Keywords:** FaABI1, FaMRLK47, FERONIA, fruit quality formation, strawberry (*Fragaria* × *ananassa*)

## Abstract

Ripening of fleshy fruits is controlled by a series of intricate signaling processes. Here, we report a FERONIA/FER-like receptor kinase, *Fa*MRLK47, that regulates both strawberry (*Fragaria* × *ananassa*) fruit ripening and quality formation. Overexpression and RNAi-mediated downregulation of *FaMRLK47* delayed and accelerated fruit ripening, respectively. We showed that FaMRLK47 physically interacts with FaABI1, a negative regulator of abscisic acid (ABA) signaling, and demonstrated that FaMRLK47 regulates fruit ripening by modulating ABA signaling, a major pathway governing strawberry fruit ripening. In accordance with these findings, overexpression and RNAi-mediated downregulation of *FaMRLK47* caused a decrease and increase, respectively, in the ABA-induced expression of a series of ripening-related genes. Additionally, overexpression and RNAi-mediated downregulation of *FaMRLK47* resulted in an increase and decrease in sucrose content, respectively, as compared with control fruits, and respectively promoted and inhibited the expression of genes in the sucrose biosynthesis pathway (*FaSS* and *FaSPS)*. Collectively, this study demonstrates that FaMRLK47 is an important regulator of strawberry fruit ripening and quality formation, and sheds light on the signaling mechanisms underlying strawberry fruit development and ripening.

## Introduction

Fleshy fruits are physiologically classified as climacteric or non-climacteric. Climacteric fruits show a sharp increase in respiration during the ripening process, while non-climacteric fruits do not (Nitsch, 1953; Coombe, 1976; Brady, 1987). Most basic studies of fruit development and ripening have focused on climacteric fruits, such as the model plant *Solanum lycopersicum* (tomato). *Fragaria* × *ananassa* (strawberry) is a typical non-climacteric fruit. Studies of strawberry fruit development and ripening are likely to provide insight into the regulatory mechanisms underlying non-climacteric fruit development and ripening.

The ripening of fleshy fruits is a complex process involving dramatic changes in physiological and biochemical metabolism, which trigger changes in color, texture, flavor, and aroma (Giovannoni, 2001; Seymour et al., 2013). In the past decades, studies of fruit ripening have mainly focused on these metabolic changes, particularly regarding their interactions with phytohormones. Ehylene has long been known to be the critical signal controlling ripening of climacteric fruits (Nitsch, 1953; Coombe, 1976; Brady, 1987; Klee and Giovannoni, 2011). Early studies suggested that auxin (IAA) is a key regulator of strawberry fruit growth and ripening (Veluthambi and Poovaiah, 1984; Given et al., 1988). Whereas overexpression of *FaNCED1*, which encodes a key enzyme in the abscisic acid (ABA) biosynthesis pathway, promoted strawberry fruit ripening, knock-down of this gene delayed it Jia et al. (2011). Furthermore, manipulating the expression of an ABA receptor, FaPYR1, and of its downstream signal members, ABI1 and SnRK2.6, affected the accumulation of anthocyanins and other fruit qualities (Chai et al., 2011; Jia et al., 2013a; Han et al., 2015). These studies suggested that ABA is an important signal controlling strawberry fruit ripening. Besides IAA and ABA, there is evidence that ethylene (White, 2002; Trainotti et al., 2005; Villarreal et al., 2010) and jasmonic acid (Concha et al., 2013) also regulate strawberry fruit ripening and quality formation. Collectively, it appears that strawberry fruit development and ripening are regulated by the synergistic effects of multiple phytohormones. We recently showed that sucrose also regulates anthocyanin accumulation in strawberry fruit (Jia et al., 2013b).

While the senescence-associated, deteriorative aspects of ripening have historically been emphasized, it is now commonly accepted that ripening is a complex process determined by a series of signaling events (Brady, 1987; Fischer and Bennett, 1991; Giovannoni, 2001). At the cellular level, the process spanning fruit set to ripening can be categorized into three major stages: cell division, cell differentiation and expansion, and cell degradation (Nitsch, 1953; Giovannoni, 2001; Seymour et al., 2013). Regulation of the cell wall’s physical properties is essential for plant growth and development, and a cell wall signaling pathway that reports on the status of the cell wall has long since been predicted to exist (Wolf et al., 2012). Recently, plasmalemma-anchored receptor-like kinases (RLKs) have attracted much attention due to their roles in sensing cell wall integrity (Humphrey et al., 2007; Cheung and Wu, 2011; Boisson-Dernier et al., 2011; Lindner et al., 2012). RLKs constitute a gene subfamily of over 600 members in *Arabidopsis* (Shiu and Bleecker, 2001a,b, 2003). Malectin is a membrane-anchored protein of the endoplasmic reticulum that recognizes and binds to Glc2-N-glycan, thereby regulating the production and secretion of *N*-glycosylated proteins (Schallus et al., 2008, 2010; Takeda et al., 2014). Interestingly, a group of RLKs harbors an extracellular sequence with a unique domain that is similar to malectin (Schulze-Muth et al., 1996; Boisson-Dernier et al., 2011; Lindner et al., 2012). The first malectin domain-containing RLK, CrRLK1, was identified in *Catharanthus roseus.* The *Arabidopsis thaliana* genome contains 17 *CrRLK1*-like *RLK* genes (Schulze-Muth et al., 1996), several of which have been functionally identified. FERONIA (FER) belongs to the CrRLK1-like subfamily and was first identified for its role in fertilization (Huck et al., 2003). FER directly interacts with guanine nucleotide exchange factors (RopGEFs), which activate downstream components that mediate the production of reactive oxygen species (ROS) at the entrance point of the female gametophyte, thereby inducing pollen tube rupture and sperm release (Duan et al., 2014; Ngo et al., 2014; Kessler et al., 2015). Furthermore, FER is a pivotal mediator of cross-talk between phytohormones, including ABA, brassinosteroids (BRs), and ethylene (Yu et al., 2012). The mechanism by which FER modulates ABA signaling was revealed in a study by Yu et al. (2012), which found that ROP11, a downstream component of FER signaling, physically interacts with ABI2, a key signal in the ABA signaling pathway. As described above, IAA, ABA, BR, and ethylene are all critical regulators of strawberry fruit development and ripening. Although *Arabidopsis* is intrinsically different from strawberry with respect to fruit development and ripening, the convergent roles of FER in phytohormone signaling prompted us to examine whether FER-like kinase is involved in strawberry fruit development and ripening. Besides FER, a few of the other malectin domain-containing RLKs have also been functionally characterized in *Arabidopsis*. Theseus1 (THE1), which was identified in a screen for suppressors that attenuated the short hypocotyl phenotype of dark-grown seedlings, functions as a cell wall integrity sensor that mediates the disruption of cellulose synthesis (Hematy et al., 2007; Hematy and Hofte, 2008; Boisson-Dernier et al., 2011). Anxur1 (ANX1) and Anxur2 (ANX2), two close relatives of FER, were also found to be pollen-specific and to regulate pollen tube rupture and sperm release (Boisson-Dernier et al., 2009). As a redundant homolog of THE1, HERCULES 1 (HERK1) was demonstrated to regulate plant growth and development (Guo et al., 2009a,b).

Malectin domain-containing RLKs (MRLKs) have been proposed to sense cell wall integrity and FER, a member of the malectin domain-containing RLK family, has been implicated in phytohormone cross-talk (Duan et al., 2010; Huang et al., 2013; Chen et al., 2016). We identified 62 MRLK members in strawberry, which we named *FaMRLK1* to *FaMRLK62* based on their chromosome location (Zhang et al., 2016). In this study, we found that FaMRLK47, the homolog of FER, is a negative regulator of strawberry fruit development and ripening. Overexpression and RNAi-mediated downregulation of *FaMRLK47* delayed and accelerated fruit ripening, respectively. FaMRLK47 physically interacts with FaABI1, and regulates fruit ripening by modulating ABA signaling, which results in changes in fruit ripening and qualities, such as sugar content and pigment accumulation. These findings provide insights into the molecular basis for the regulation of strawberry fruit development and ripening.

## Materials and Methods

### Plant Materials and Growth Conditions

Strawberry plants (*Fragaria × ananassa* ‘Benihoppe’) were grown on soil supplemented with nutrient soil, organic fertilizer, and vermiculite (7:2:1; v/v/v) in the greenhouse. The controlled condition of greenhouse was 12/12-h photoperiod at 450 μmol m^-2^ s^-1^ and 70% humidity, under day/night temperature of 25°C/15°C.

### Gene Isolation and Sequence Analysis

The cDNA sequences of full-length RLKs were obtained from the TAIR website^1^ and NCBI^2^. To identify *CrRLK1-like RLK* (*CrRLK1L*) genes in strawberry, the coding sequence of *FERONIA* (*At3g51550*) was used as query to BLAST the strawberry genome. Phylogenetic trees were constructed using the Neighbor-Joining (NJ) method in MEGA 4.0.2 software, with 1000 bootstrap replicates to evaluate the reliability of different phylogenetic groups. The deduced amino acid sequences of FaMRLKs were aligned using ClustalX 2.0.12 with default settings. The alignments were edited and marked using GeneDoc.

To isolate *FaMRLK47* and *FaMRLK50*, total RNA was extracted from strawberry fruit using an E.Z.N.A.^®^Total RNA Kit (OMEGA). The cDNA was synthesized from 1 μg of total RNA using M-MLV Reverse Transcriptase (Promega) according to the manufacturer’s instruction. Full-length *FaMRLK47* and *FaMRLK50* were cloned by RT-PCR from cDNA using Q5 High-Fidelity DNA Polymerase (New England Biolabs) under the following conditions: 94°C/30 s for 1 cycle, 94°C/30 s, 56°C/25 s, and 72°C/5 min for 35 cycles, and a final extension of 72°C/5 min. The amplified fragments were subcloned into the pMD19-T vector and transformed into *Escherichia coli DH5α*. Then the selected positive colonies were sequenced by Invitrogen to confirm the full-length sequence. The Primer sequences and GenBank accession numbers are shown in Supplementary Table S1.

### Quantitative Reverse Transcriptase PCR (RT-qPCR)

Quantitative reverse transcriptase PCR (RT-qPCR) was performed using SYBR Premix Ex TaqTM (TaKaRa) in a ABI7500 Real-Time PCR System. Primers used for RT-qPCR were designed using Primer3 Plus^3^. Three biological replicates were set up, and each sample was analyzed at least in triplicate. *FaACTIN* was used as an internal control and the 2^-ΔΔCT^ method (where ΔCT represents the difference between the cycle threshold values of the target and reference genes) was used to calculate the relative transcript levels (Schmittgen and Livak, 2008).

#### Plant Material and Treatments

The process from fruit set to ripening was classified into six stages as follows: small green fruit (abbreviated as SG), large green fruit (LG), white fruit (W), initially reddening (IR), and fully reddening (FR). For each stage, five fruits were combined as an individual sample. After fruits were frozen in liquid nitrogen, seeds (achenes) were removed with a needle, and the receptacles were used to analyze gene expression. The expression of *FaMRLKs* was assessed by RT-qPCR analysis, using the primer sequences shown in Supplementary Table S2.

For phytohormone treatment, fruit disks (10 mm in diameter and 1 mm in thickness) were prepared and combined from 20 fruits in the large green stage. For each treatment, disk samples (5 g per sample) were equilibrated for 30 min in equilibration buffer (Archbold, 1988; 10 mM MgCl_2_, 5 mM CaCl_2_, 200 mM mannitol, 10 mM EDTA, 5 mM vitamin C, and 50 mM MES-Tris, pH 5.5) and then shaken for 6 h at 25°C in equilibration buffer containing 100 μM ABA or 200 μM IAA under darkness. After a 6-h incubation, the samples were washed with deionized water, frozen immediately in liquid nitrogen, and kept at -80°C until use. For temperature treatment, the fruit was split longitudinally into two even parts; one half was subjected to high (40°C, 8 h) or low (4°C, 24 h) temperature treatment, and the other (the control) was incubated for the same period at 25°C. After treatment, the fruits without seeds were frozen in liquid nitrogen and stored at -80°C until use. Each individual analysis was conducted in triplicate. Primers of ripening-related genes used for the RT-qPCR analysis are presented in Supplementary Table S3.

### Transfection of Strawberry by Agroinfiltration and ABA Treatment

To construct vectors for overexpression of *FaMRLK47* and *FaMRLK50* (abbreviated hereafter as *FaMRLK47-OE* and *FaMRLK50-OE*), full-length *FaMRLK47* and *FaMRLK50* were cloned into the plant expression vector pCambia1304 using the *XbaI* and *SacI* restriction sites. To construct vectors for downregulating *FaMRLK47* (abbreviated hereafter as *FaMRLK47-RNAi*), the plant expression vector pFGC5941 was used. pCambia1304, pFGC5941, *FaMRLK47*-OE, *FaMRLK47*-RNAi, and *FaMRLK50*-OE were transformed individually into *Agrobacterium tumefaciens* strain EHA105 (Lazo et al., 1991). The transformed strains were grown at 28°C in Luria-Bertani liquid medium containing 10 mM MES and 20 μM acetosyringone with appropriate antibiotics. When the culture reached an optical density at 600 nm of approximately 0.8, *A. tumefaciens* cells were harvested, resuspended in infection buffer [10 mM MgCl_2_, 10 mM MES (pH 5.6), and 200 mM acetosyringone], and shaken for 2 h at room temperature before being used for infiltration. Pairs of fruits at 18 DPA (day past anthesis) and with similar phenotypes were selected and, for each pair of fruits, one was transfected with *FaMRLK47*-OE, *FaMRLK50*-OE, or *FaMRLK47*-RNAi and the other (the control) was transfected with pCambia1304, pCambia1304 empty vector, or pFGC5941, respectively. For transfection, *A. tumefaciens* suspension was evenly injected into the fruits with a syringe until the whole fruit became hygrophanous (Jia et al., 2013a), and for each gene, 25 pairs of fruit were injected. To examine the expression of ripening-related genes, *FaMRLK47*-OE and *FaMRLK50*-OE transformed fruits were collected 12 days after infiltration and *FaMRLK47*-RNAi fruit was collected 8 days after infiltration. After removing seeds, fruit samples were frozen in liquid nitrogen and kept at -80°C until used. To investigate the effect of *FaMRLK47*-OE or *FaMRLK47*-RNAi on ABA signaling in fruits, detached fruits were transfected with the *FaMRLK47*-OE, *FaMRLK47*-RNAi, or empty pCambia1304 vector, and then incubated at 22°C and 100% humidity. Three days after the transfection and incubation, fruits were treated with 100 μM ABA for 6 h and expression of the selected genes was analyzed as described above.

### Determination of Fruit Ripening-Associated Physiological Parameters and ABA Content

Flesh firmness was measured after removing fruit skin on opposite sides of the fruit using a GY-4 fruit hardness tester (Zhejiang Top Instrument). The contents of anthocyanins, flavonoid, and total phenol in the fruit were evaluated using described methods (Fuleki and Francis, 1968; Lees and Francis, 1971). The soluble sugar content was examined as described by Jia et al. (2011). The total titratable acidity calculated, expressed as percent malic acid, was measured using the acid–base titration method (Kafkas et al., 2007). Volatile organic components were analyzed by headspace solid-phase microextraction and gas chromatography–mass spectrometry as described by Dong et al. (2013). ABA content was measured by an indirect enzyme-linked immunosorbent assay (ELISA) (Zhang et al., 2009). The ELISA procedures were performed according to the instructions provided by the manufacturer (China Agricultural University, Beijing, China) and the assay plates were read by the Thermo Electron (Labsystems) Multiskan MK3 (PIONEER, Co., Beijing).

### Yeast Two-Hybrid Assays

Yeast two-hybrid assays were performed using the Matchmaker GAL4-based Two-Hybrid System 3 (Clontech), according to the manufacturer’s instructions. The coding sequence of the FaMRLK47 kinase domain (540–892 aa) was fused in-frame with the GAL4 DNA-binding domain (BD) in the pGBKT7 vector to generate the FaMRLK47-BD plasmid. The full-length cDNA sequences of *FaABI1* were inserted into the pGADT7 vector. Four different combinations, pGADT7/pGBKT7, FaABI1-AD/pGBKT7, pGADT7/FaMRLK47-BD, and FaABI1-AD /FaMRLK47-BD were respectively transformed into AH109 strains using the lithium acetate method. After transfection, strains were steaked on -Leu/-Trp medium and further selected on minimal -Leu/-Trp/-His/-Ade medium, and then treated with 20 μg/mL X-Gal for interaction validation. Combinations of pGADT7/pGBKT7, FaABI1-AD/pGBKT7, and pGADT7/FaMRLK47-BD were used as negative controls. The primers used for yeast two-hybrid assays are provided in Supplementary Table S4.

### Bimolecular Fluorescence Complementation (BiFC) and Subcellular Localization Assays

For the bimolecular fluorescence complementation (BiFC) assay, the full-length cDNA sequence of *FaMRLK47* or *FaABI1* was cloned into pCambia1300-YFP^n/c^ to generate the interaction vectors FaMRLK47-YFP^c^ or FaABI1-YFP^n^. FaMRLK47-YFP^c^ or FaABI1-YFP^n^ plasmids were further transformed into *A. tumefaciens* strain EHA105, and cultured at 28°C. To examine the *in vivo* interaction, FaMRLK47-YFP^c^ and FaABI1-YFP^n^ were co-expressed in tobacco leaves (*Nicotiana tabacum*) by *Agrobacterium*-mediated infiltration (Schütze et al., 2009). The negative control was performed by co-expressing FaMRLK47-YFP^c^ and empty pCambia1300-YFP^n^ vector in tobacco leaves. Chimeric fluorescence was examined by confocal microscopy (Olympus Fluoview FV1000). For YFP and bright field imaging, excitation wavelengths of 488 and 543 nm were used, respectively.

For subcellular localization of FaMRLK47, full-length cDNA sequences were amplified by PCR using the forward primer 5′-TTAATTAAATGAAGTGTTTCTTTTTCTATATTTGGTTC-3′ and the reverse primer 5′-GGCGCGCCAACGTCCCTTTGGGTTCATGATTTGTGAG-3′. The PCR fragments were inserted into pMDC83 using *Asc1* and *Pac1* and the constructs were then introduced into *A. tumefaciens* strain EHA105 and transformed into tobacco leaves as described by Schütze et al. (2009). Transfected plants were grown in darkness for 24 h and in light for 48 h at 24°C. After 3 days, fluorescence was observed using a confocal laser-scanning microscope (Olympus Fluoview FV1000). The primers used for BiFC and localization are shown in Supplementary Table S5.

### Statistical Analysis

Samples were analyzed in triplicate, and the data were noted as the mean ± SD. Data were analyzed using Student’s *t*-test implemented in SAS software (version 8.1, United States), and the least significant difference at a 0.05 level of probability was used to explore the effect of P input on parameters. A *P*-value of ≤ 0.05 was considered to indicate a significant difference, and a *P*-value of ≤ 0.01 was considered to indicate a highly significant difference.

## Results

### Genome-Wide Identification of *FER*-Like Genes

**FIGURE 1:**
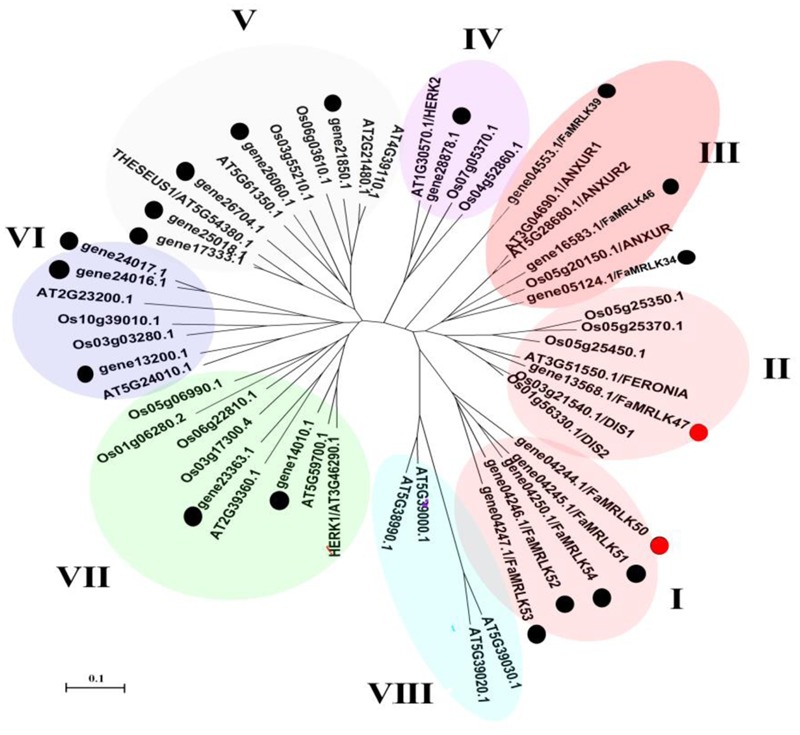
Phylogenetic tree of deduced FaMRLK amino acid sequences. Phylogenetic analysis of CrRLK1L homologs from various plant species. The phylogeny was constructed using the Neighbor-Joining method in MEGA4.0.2 with 1,000 bootstrap replicates. FER-like proteins in strawberry are marked with black circles. At, *Arabidopsis thaliana*; Os, *Oryza sativa*.

The FERONIA-like genes belong to a family of CrRLK1-like RLKs (CrRLK1Ls), which in turn belong to a super-family of malectin domain-containing RLKs. In a previous study (Zhang et al., 2016), we conducted a genome-wide screen of woodland strawberry, *Fragaria vesica*, for ‘Malectin domain-containing RLKs’ (accordingly designated as MRLKs) and identified 62 members (named FvMRLK1–62). In the present study, a screen of the *F. vesca* genome revealed a CrRLK1L family consisting of 20 members. Phylogenetic analysis of CrRLK1L homologs from various plant species revealed eight clades, with FERONIA, NAXURs, HERKs, and THESEUSs being distributed in different clades (**Figure 1** and Supplementary Figure S1). Notably, FERONIA was located in Clade II and only one member of the FaCrRLK1L family, i.e., FaMRLK47, was located in this clade. FaMRLK47 showed a relatively high level of amino acid sequence identity (72.33%) with FERONIA, and can thus be viewed as a homolog of FER. Interestingly, clade I, which contains five members (FaMRLK50–54), was found to consist exclusively of proteins from the strawberry genome. Furthermore, members of clades I, II, and III exhibited relatively high levels of amino acid sequence identity with FERONIA (FER hereafter), and can thus be considered FEL-like receptor kinases. FaCrRLK1Ls and FER exhibited between 42.45 and 48.03% amino acid sequence identity for clade I proteins and between 38.02 and 50.66% for clade III proteins.

### Expression Profile of the *FER*-Like Receptor Kinases during Strawberry Fruit Development and Ripening

**FIGURE 2:**
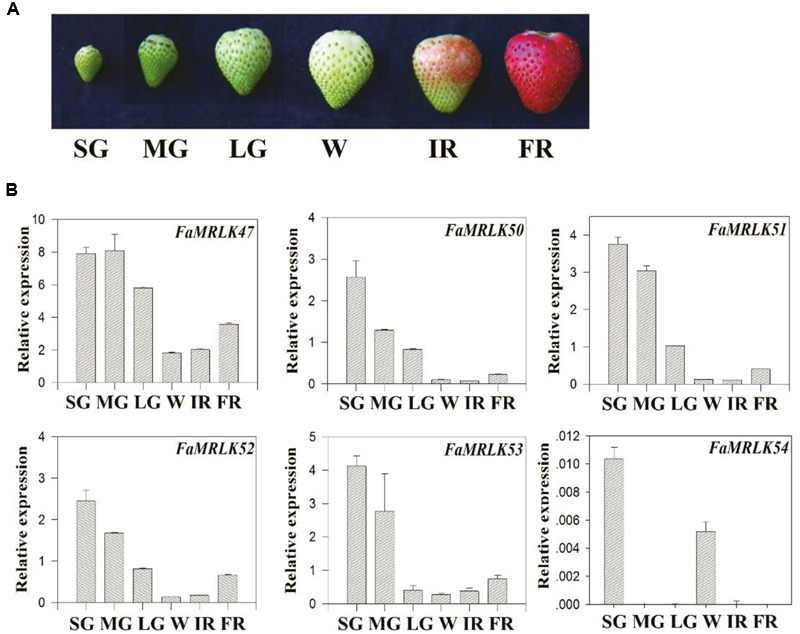
Temporospatial pattern of *FaMRLK* expression in strawberry fruits. **(A)** Phenotypes of strawberry fruit at different developmental stages: small green (SG), middle green (MG), large green (LG), white (W), initial reddening (IR), and fully reddening (FR). **(B)** Quantitative reverse transcriptase PCR (RT-qPCR) analysis of the expression of various *FaMRLK* genes at the indicated developmental stages. Labels below bars denote the corresponding developmental stages, as in **(A)**. *FaACTIN* was used as an internal control. Values are means ± SD of three biological replicates.

Strawberry fruit development, from fruit set to ripening, can be divided into several substages, i.e., the small green fruit (SG), middle green fruit (MG), large green fruit (LG), white fruit (W), initially reddening fruit (IR), and fully reddening fruit (FR) substages (**Figure 2A**). Given that FaMRLK47 is a homolog of FER and the only FaMRLK member in clade II (**Figure 1**), we focused on this protein in the present study. As the members of clade I only existed in the strawberry genome and members of clade II exhibited relatively high levels of amino acid sequence identity with FERONIA, we also evaluated their expression in relation to strawberry fruit development and ripening (**Figure 2B**). Whereas *FaMRLK34, FaMRLK39*, and *FaMRLK6* transcripts were not detected in strawberry fruit, the relative levels of the other six members differed, all tending to decrease from the SG to W substages. Notably, *FaMRLK47* expression was higher than that of other members from clade I and II. Furthermore, while the expression levels of *FaMRLK47* started to drop during the MG substage, those of all other members examined started to decline during the SG substage, which suggests that *FaMRLK47* is more tightly associated with the onset of fruit ripening.

### Expression of the *FER*-Like Receptor Kinases in Response to Internal and Environmental Signals Involved in the Regulation of Strawberry Fruit Development and Ripening

**FIGURE 3:**
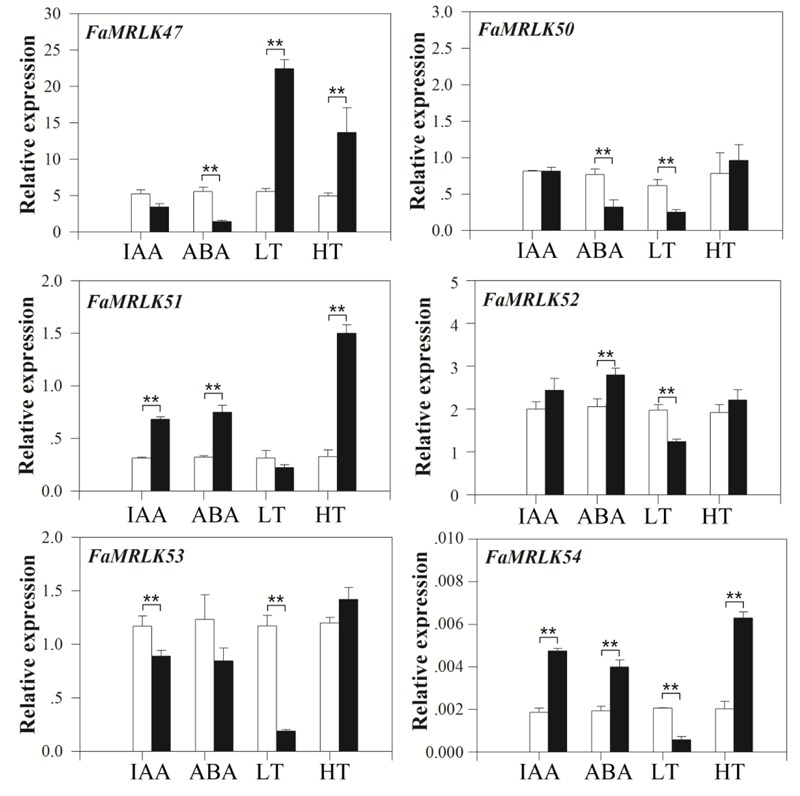
Quantitative reverse transcriptase PCR analysis of *FaMRLK* expression in response to IAA, ABA, low temperature (LT), and high temperature (HT) treatments in fruits at the LG stage. RT-qPCR was conducted using *FaACTIN* as an internal control. Values are means ± SD of three biological replicates. Asterisks denote significant differences compared with the control sample (i.e., the 0 concentration for hormone treatment and 25°C for temperature treatment) at ^∗^*P* < 0.05 and ^∗∗^*P* < 0.01, according to Student’s *t*-test. White bars indicate control samples; black bars indicate treated samples.

Strawberry fruit development and ripening are regulated by both internal and external factors, including IAA, ABA, and temperature (Giovannoni, 2001; Seymour et al., 2013). While the application of IAA delays strawberry fruit ripening, application of ABA promotes it Given et al. (1988) and Jia et al. (2011, 2013a). In our previous studies, we found that ripening of strawberry fruit was stimulated by high temperatures and delayed by low temperatures (Han et al., 2015). We therefore studied the expression profiles of the *FER*-like *FaMRLKs* in response to IAA, ABA, and low/high temperature treatment. While the transcription of all of these genes was sensitive to IAA, ABA, and temperature treatments, their responses differed. *FaMRLK47, FaMRLK50*, and *FaMRLK53* expression were inhibited by ABA treatment, whereas *FaMRLK51* and *FaMRLK52* expression were upregulated by ABA treatment. *FaMRLK47, FaMRLK50, FaMRLK52*, and *FaMRLK53* expression were also sensitive to temperature stress; however, while *FaMRLK47* expression was promoted by both low and high temperature treatment, *FaMRLK52* expression was inhibited and promoted, respectively, by low and high temperature treatments (**Figure 3**).

### Manipulating *FaMRLK47* Expression Caused Changes in the Progress of Fruit Development and Ripening

**FIGURE 4:**
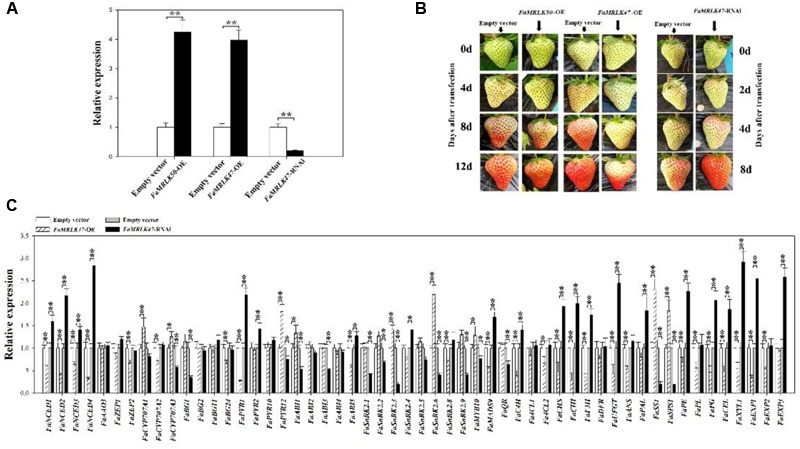
Effect of *FaMRLK47* or *FaMRLK50* overexpression (OE) and RNAi-mediated silencing of *FaMRLK47* on strawberry (*Fragaria* × *ananassa*) fruit ripening. **(A)** RT-qPCR analysis of *FaMRLK47* and *FaMRLK50* expression in OE, RNAi, and control fruits. *FaMRLK47* was overexpressed or silenced, and *FaMRLK50* was overexpressed as described in Section “Materials and Methods.” The overexpression or RNAi constructs were injected into the fruits at 18 DPA and gene expression was analyzed 12 days after transfection with the OE vector or 8 days after transfection with the RNAi vector. Control samples were transfected with the empty vector (pCambia1304). RT-qPCR was conducted using *FaACTIN* as an internal control. Values are means ± SD of three biological replicates. Double asterisks denote significant difference at *P* < 0.01 using Student’s *t*-test. **(B)** The influence of *FaMRLK47-*OE, *FaMRLK47-*RNAi, and *FaMRLK50*-OE on the time course of strawberry fruit development and ripening. **(C)** The effect of *FaMRLK47-*OE and *FaMRLK47-*RNAi on the expression of ripening-related genes. Values are means ± SD of three biological replicates. Asterisks denote significant differences compared with the control sample (i.e., Empty vector) at ^∗^*P* < 0.05 and ^∗∗^*P* < 0.01, using Student’s *t*-test.

To investigate a potential role of *FaMRLK47* in the regulation of strawberry fruit development and ripening, we transiently manipulated its expression in strawberry fruits. As a representative member of the *FaMRLK* family that specifically exists in the strawberry genome, *FaMRLK50* was also investigated. Since *FaMRLK47* and *FaMRLK50* transcript levels dramatically decreased from the SG to LG substages, we first sought to examine the function of *FaMRLK47* and *FaMRLK50* by transiently overexpressing these two genes in strawberry plants. As shown in **Figure 4A**, overexpression of *FaMRLK47* and *FaMRLK50* resulted in a great increase in their transcript levels in fruits. While overexpression of *FaMRLK50* did not affect fruit development and ripening, overexpression of *FaMRLK47* delayed fruit ripening, as reflected by pigment accumulation. Conversely, RNAi-mediated downregulation of *FaMRLK47* accelerated fruit ripening (**Figures 4A,B**). Collectively, these experiments indicate that *FaMRLK47* is an important regulator of strawberry fruit development and ripening.

**FIGURE 5:**
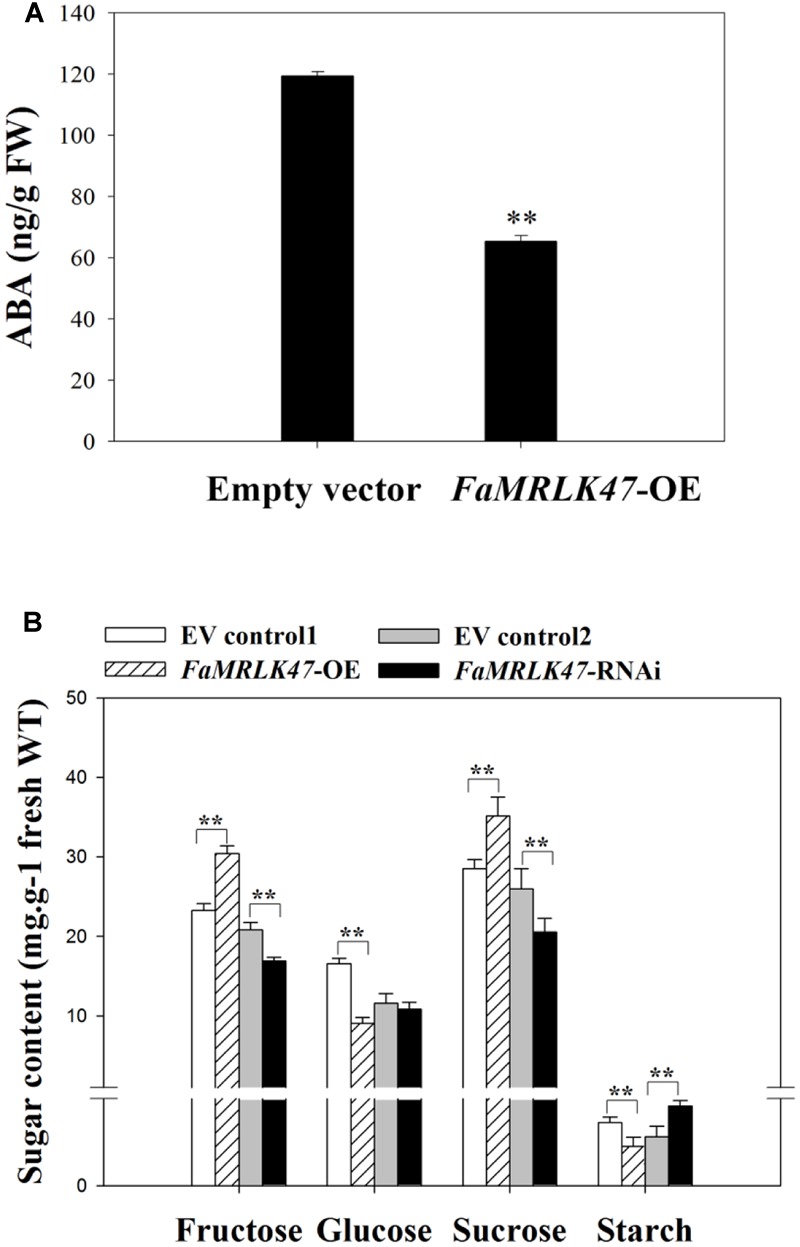
Changes in ABA content and sugar metabolism during strawberry fruit growth and development. **(A)** Changes of ABA content in *FaMRLK47*-OE fruits. Values are means ± SD of three biological replicates. Asterisks denote significant differences compared with the control sample (i.e., OE-C) at ^∗∗^*P* < 0.01, using Student’s *t*-test. **(B)** Changes in sugar metabolism during fruit development (glucose, open circles; fructose, open squares; sucrose, open triangles). Values are means ± SD of three samples. ^∗∗^*P* < 0.01 (Student’s *t*-test), when compared with control values.

To further explore the *FaMRLK47*-mediated mechanisms underlying the regulation of strawberry fruit development and ripening, we examined the effects of *FaMRLK47* overexpression and downregulation on the expression of a series of ripening-related genes (**Figure 4C**). Most of these ripening-related genes are important structure genes and transcription factors that are involved in the formation of fruit qualities such as color, texture, aroma, and sugar (Jia et al., 2011, 2013a; Seymour et al., 2011; Lin-Wang et al., 2014; Han et al., 2015). Given that fruit ripening is highly affected by ABA, we also detected the expression of genes involved in ABA biosynthesis, metabolism, and signal transduction. As shown in **Figure 4C**, manipulating *FaMRLK47* expression altered the expression patterns of both ripening-related and ABA-related genes, which implied that FaMRLK47 is an important regulator of diverse processes in fruit ripening, including fruit quality formation, ABA production, and signal transduction. Further detection of the ripening-related physiological parameters demonstrated that overexpression and downregulation of *FaMRK47* resulted in a decrease and increase, respectively, in most of the fruit quality parameters that were expected to increase and decrease during fruit development and ripening (**Table 1**). Moreover, the ABA content showed a decline in *FaMRLK47*-OE fruit (**Figure 5A**). Comprehensive analysis of the changing patterns of gene expression and physiological parameters showed that *FaMRLK47* mainly plays a role in the regulation of anthocyanins accumulation, flavonoid metabolism and fruit softness, and *FaCHS, FaCHI, FaUFGT, FaPAL, FaPE, FaPG, FaXYL1*, and *FaEXP1* might function as the important downstream structure genes of *FaMRLK47* in these processes. In addition, as shown in **Figure 4C**, *FaNCED1-4, FaCYP707A, FaPYL1/12, FaABI1/5*, and *FaSnRK2.3/2.6* might participate in *FaMRLK47*- mediated ABA production and signal transduction.

**Table 1:** Effects of *FaMRLK47*-OE and *FaMRLK47*-RNAi on major fruit ripening-related parameters.

Parameters	OE-C	OE	RNAi-C	RNAi	Notes
Firmness (kg.cm^-2^)	3.15 ± 0.776	7.375 ± 1.563**	6.435 ± 0.172	2.335 ± 0.168**	Cell wall metabolism-related parameter
Flavonoid content (μg.g^-1^.fresh wt)	2.309 ± 0.035	3.516 ± 0.102**	3.968 ± 0.106	2.774 ± 0.037**	Pigment metabolism-related compounds
Anthocyanin content (mg.g^-1^.fresh wt)	0.78 ± 0.006	0.07 ± 0.003**	0.106 ± 0.008	0.652 ± 0.006**	
Total phenol content (μg.g^-1^.fresh wt)	7.382 ± 0.054	5.74 ± 0.069**	6.885 ± 0.075	9.65 ± 0.105**	
Total titratable acid content (%)	2.488 ± 0.036	3.104 ± 0.123**	4.115 ± 0.0562	1.788 ± 0.065**	Acid metabolism-related parameter
Acetic acid, methyl ester	4.24 ± 0.015	4.27 ± 0.008	3.765 ± 0.122	4.826 ± 0.365	Aroma metabolism-related compounds (expressed as percentage of the total volatiles)
Acetic acid, 1-methylethyl ester	0.155 ± 0.078	0.425 ± 0.098**	0.388 ± 0.006	0.000 ± 0.000**	
Silanediol, dimethyl	0.63 ± 0.011	0.85 ± 0.024	0.664 ± 0.115	0.365 ± 0.018**	
Butanoic acid, methyl ester	1.245 ± 0.163	1.01 ± 0.102	0.998 ± 0.086	0.906 ± 0.102	
2-Pentenal (E)	0.095 ± 0.002	0.125 ± 0.007**	0.88 ± 0.076	0.105 ± 0.008**	
Butanoic acid, 3-methyl-, methyl ester	0.13 ± 0.004	0.56 ± 0.012**	0.000 ± 0.000	0.105 ± 0.053**	
Hexanal	17.27 ± 2.931	18.95 ± 1.281*	15.535 ± 3.096	16.778 ± 1.055	
2-Hexenal (E)	0.88 ± 0.024	1.48 ± 0.099**	0.955 ± 0.076	0.35 ± 0.006**	
1-Butanol, 2-methyl-, acetate	0.08 ± 0.005	0.84 ± 0.016**	0.000 ± 0.000	0.000 ± 0.000	
Hexanoic acid, methyl ester	1.62 ± 0.605	4.055 ± 0.142**	2.455 ± 0.205	1.218 ± 0.08	
2-Heptenal (Z)	0.08 ± 0.095	0.23 ± 0.014**	0.000 ± 0.000	0.000 ± 0.000	
Hexanoic acid	2.01 ± 0.042	1.035 ± 0.077*	0.000 ± 0.000	0.785 ± 0.006**	
Octanal	0.135 ± 0.063	0.255 ± 0007*	0.000 ± 0.000	0.000 ± 0.000	
3(2H)-Furanone, 4-methoxy-2, 5-dimethyl	2.99 ± 0.159	0.445 ± 0.017**	0.694 ± 0.022	4.519 ± 0.03**	
Hexanoic acid, 2-oxo-, methyl ester	0.6 ± 0.004	0.46 ± 0.028**	0.236 ± 0.052	0.382 ± 0.105	
1-Octanol	0.205 ± 0.049	0.24 ± 0.042	0.198 ± 0.006	0.000 ± 0.000**	
1,6-Octadien-3-ol, 3,7-dimethyl	0.96 ± 0.035	0.99 ± 0.117	0.78 ± 0.006	0.422 ± 0.078**	
Nonanal	0.215 ± 0.064	0.36 ± 0.042*	0.215 ± 0.09	0.382 ± 0.078	
Phenol,2,4-*bis*(1,1-dimethylethyl)	0.19 ± 0.042	0.275 ± 0.021*	0.384 ± 0.008	0.096 ± 0.002**	
Dodecanoic acid, ethyl ester	0.075 ± 0.007	0.115 ± 0.021**	0.215 ± 0.065	0.302 ± 0.096	

*The *FaMRLK47*-OE or *FaMRLK47*-RNAi construct was transfected into fruits at 18 DPA. Fruits were detached and analyzed 12 or 8 days after transfection. Values are means ± SD of two samples (each sample includes five fruits). Asterisks denote significant differences compared with the control sample (i.e., OE-C or RNAi-C) at *P* < 0.01, using Student’s *t*-test.*

Interestingly, in contrast to the changes in expression profiles of other ripening-related genes following *FaMRLK47* manipulation, overexpression and downregulation of *FaMRLK47* respectively promoted and inhibited the expression of *FaSS* and *FaSPS1*, two key genes in the sucrose biosynthesis pathway (**Figure 4C**). Further analysis of sugar metabolism showed that, while RNAi-mediated downregulation of *FaMRLK47* expression resulted in a dramatic increase in the major sugar components of the fruit, i.e., sucrose, fructose and glucose, overexpression of *FaMRLK47* caused a significant decrease in sucrose and fructose content (**Figure 5B**). Additionally, overexpression and RNAi-mediated downregulation of *FaMRLK4*7 resulted in a significant decrease and increase in starch content, respectively. Collectively, these results suggest that FaMRLK47 is an important regulator of sugar metabolism.

FaMYB10 was reported to be an important positive regulator of anthocyanin accumulation, whereas FaMADS9 was shown to regulate fruit development and ripening (Seymour et al., 2011; Lin-Wang et al., 2014). In this study, we also monitored the expression of *FaMYB10* and *FaMADS9* (**Figure 4C**), and found that the expression of *FaMADS9* was repressed in *FaMRLK47*-OE fruit and upregulated in *FaMRLK47*-RNAi fruit. Although *FaMYB10* expression was not expected to be altered by changes in *FaMRLK47* expression, overexpression of *FaMRLK47* resulted in an increase in *FaMYB10* expression, and downregulation of *FaMRLK47* resulted in a decrease in *FaMYB10* expression. These results imply that FaMRLK47 and FaMYB10 are regulatory proteins with diverse functions in fruit ripening. Furthermore, their regulatory mechanisms are more complex than previously expected.

### Manipulation of *FaMRLK47* Expression Modifies the Expression of ABA-Induced Genes in Fruit

**FIGURE 6:**
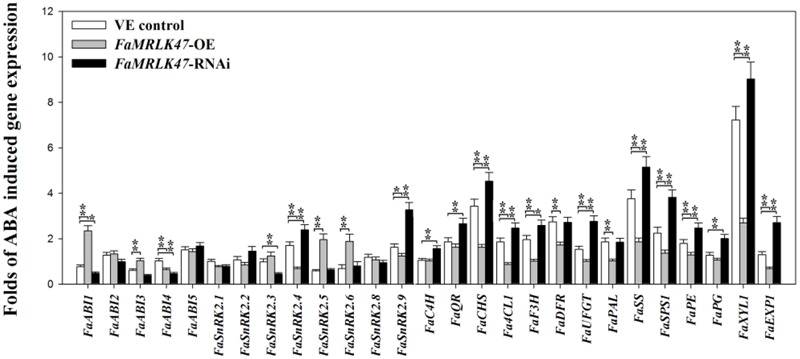
Effect of *FaMRLK47*-OE and *FaMRLK47*-RNAi on the sensitivity of ripening-related genes to ABA. Quantification of the sensitivity of ripening-related gene expression to ABA, with gene expression being expressed as a ratio of ABA treatment/non-treatment control. *FaMRLK47*-OE and *FaMRLK47*-RNAi samples were treated with or without ABA as described in Section “Materials and Methods.” Values are means ± SD of three replicates. ^∗∗^*P* < 0.01 and ^∗^*P* < 0.05 (Student’s *t*-test), when compared with control values.

In *Arabidopsis*, FER is involved in ABA signaling (Yu et al., 2012). As FaMRLK47 shares 74% amino acid sequence identity with FER, which is known to modulate ABA signaling (Yu et al., 2012), we were interested in establishing whether FaMRLK47 was associated with ABA signaling in strawberry fruit. To investigate a possible role for *FaMRLK47* in the modification of the fruit’s response to ABA treatment, we overexpressed and downregulated *FaMRLK47* in strawberry fruit for a short time (72 h), and then examined the expression of a series of ripening-related genes following ABA treatment. As shown in **Figure 6**, overexpression of *FaMRLK47* resulted in a great decrease in the ability of ABA to induce the expression of these genes in comparison with control fruits, and conversely, RNAi-mediated downregulation of *FaMRLK47* resulted in a great increase in the ability of ABA to induce the expression of these genes. These results indicate that FaMRLK47 functions as a negative regulator of the ABA signaling cascade.

### FaMRLK47 Physically Interacts with ABI1

**FIGURE 7:**
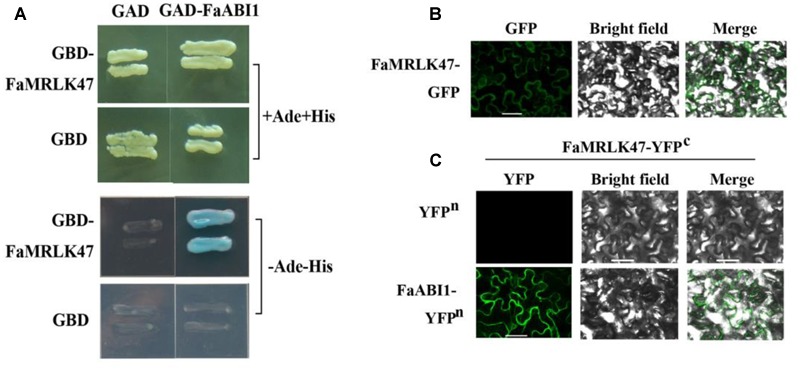
Subcellular localization of FaMRLK47 and physical interaction between FaMRLK47 and FaABI1. **(A)** Yeast two-hybrid analysis of the physical interaction between FaMRLK47 and FaABI1. Protein interactions were examined using combinations of prey and bait vectors. All tests were conducted on media containing adenine (+Ade+His; /–Leu/–Trp/+His/+Ade) or lacking adenine (–Ade–His; /–Leu/–Trp/–His/–Ade). Interactions were determined based on cell growth and were confirmed by an α-Gal assay on medium lacking adenine (/–Leu/–Trp/–His/–Ade). **(B)** Subcellular localization of FaMRLK47. pMDC83-FaMRLK47 was transformed into tobacco (*Nicotiana tabacum*) cells, and fluorescence was observed by confocal microscopy as described in Section “Materials and Methods.” Bars = 50 μm. **(C)** BiFC analysis of the physical interaction between FaMRLK47 and FaABI1. FaMRLK47 and FaABI1 were fused with the C and N terminus of yellow fluorescent protein (YFP; designated as YFPc and YFPn, respectively). Different combinations of the fused constructs were co-transformed into tobacco (*Nicotiana tabacum*) cells, and the cells were visualized using confocal microscopy as described in Section “Materials and Methods.” YFP and bright field were excited at 488 and 543 nm, respectively. Bars = 50 μm.

Given that FaMRLK47 is capable of modifying ABA signaling, we examined whether FaMRLK47 could physically interact with important signal proteins in the ABA signaling pathway. As FaABI1 is a key signaling protein in the ABA signaling pathway and negative regulator of strawberry fruit development and ripening (Jia et al., 2013a), we examined the interaction between FaMRLK47 and FaABI1. Yeast two-hybrid analysis showed that by co-transformed of FaMRLK47 and FaABI1 into AH109, the transformed strain grew well on auxotrophic medium (SD-Ade-Leu-Trp-His), which indicated that FaMRLK47 interacts with FaABI1 (**Figure 7A**). To test whether FaMRLK47 could interact with ABI1 in living plant cells, we first observed the localization of FaMRLK47 in tobacco leaf cells by fusing it with eGFP. We found that FaMRLK47 localized to the membrane in tobacco leaf cells (**Figure 7B**). Furthermore, we co-transformed the BiFC vectors FaMRLK47-YFP^c^ and FaABI1-YFP^n^ into tobacco leaves, using co-transformation of FaMRLK47-YFP^c^ and pCambia1300-YFP^n^ as a control. The results showed that, while fluorescence was not observed in the control transformed leaves, strong fluorescence appeared when FaABI1-YFP^n^ was combined with FaMRLK47-YFP^c^ (**Figure 7C**), indicating that FaABI1 and FaMRLK47 indeed physically interact.

## Discussion

Ripening is a complex process, which involves dramatic changes in physiological and biochemical metabolism, with cell wall degradation considered to be the most important event (Fischer and Bennett, 1991; Giovannoni, 2001). Cell enlargement necessitates an increase in the surface of cell walls, and cell wall extension has been assumed to take place as a result of the loosening of intercrossing cellulose fibrils (Fischer and Bennett, 1991). Cell wall loosening and subsequent material deposition and rigidification must be tightly regulated, so that cell wall integrity and plant organ development can be coordinately maintained (Humphrey et al., 2007). Therefore, deciphering the mechanisms that sense and regulate cell wall integrity is of particular importance for understanding the process of strawberry fruit development and ripening.

Receptor like protein kinases (RLKs) are important candidate sensors of cell wall integrity and cell wall metabolism. Amongst the members of the RLK family, malectin domain-containing RLKs have attracted particular interest due to the presence of an extracellular sequence of the type thought to recognize and bind to oligosaccharides (Schulze-Muth et al., 1996; Schallus et al., 2008). In *Arabidopsis*, the malectin domain-containing RLKs are proposed to be encoded by a gene subfamily, named the *CrRLK1L* family, which has 17 members (Lindner et al., 2012). Numerous studies have aimed to identify the ligands of these RLKs, particularly the oligosaccharide-like ligands (Liu et al., 2009; Wolf et al., 2012; Engelsdorf and Hamann, 2014; Haruta et al., 2014; Kessler et al., 2015). While the malectin domain implies the existence of oligosaccharide-like ligands, one cannot exclude the possibility that the malectin domain functions to anchor the protein kinase to cell walls. Therefore, one would expect cell wall degradation to affect the behavior of these RLKs. In support of this notion, the present study indicated that the expression of all malectin domain-containing RLKs dramatically decreased during fruit development and ripening (**Figure 2**), implying that malectin domain-containing RLKs are tightly associated with strawberry fruit development and ripening. Direct evidence for this came from the finding that manipulation of *FaMRLK47* expression altered the progression of fruit ripening.

FaMRLK47 shares 74% amino acid sequence identity with FER, a malectin domain-containing RLK from *Arabidopsis*. *FER* belongs to the *CrRLK1L* gene family, which consists of 17 members (Lindner et al., 2012). While little is known about most members of the CrRLK1 family, a few members have been functionally identified. FER was identified for its role in controlling pollen tube growth and fertilization (Escobar-Restrepo et al., 2007). Aside from FER, several other related members, such as THESEUS1, HERCULES, and ANXURs, have also been functionally identified (Hematy and Hofte, 2008; Miyazaki et al., 2009; Cheung and Wu, 2011). Intriguingly, studies suggest that all of these members are essentially associated with the sensing of cell wall integrity and thereby play important roles in regulating cell growth (Humphrey et al., 2007; Hematy and Hofte, 2008; Cheung and Wu, 2011; Lindner et al., 2012; Li and Zhang, 2014). Given that FaMRLK47 shares a relatively high level of amino acid sequence identity with FER and that FER-related protein kinases have been suggested to be important regulators of cell growth, it is possible that *FaMRLK47* regulates early fruit growth in addition to the onset of fruit ripening. This assumption is consistent with the pattern of *FaMRLK47* expression, i.e., the transcript levels of *FaMRLK47* remain high throughout the early stages of fruit growth and decline dramatically during veraison.

It has been reported that ABA is an important regulator of strawberry fruit ripening (Jia et al., 2011; Han et al., 2015). In *Arabidopsis*, FER-mediated ABA signaling is based on a physical interaction between FER and guanine exchange factors (GEFs) (Yu et al., 2012). Specifically, FER physically interacts with GEFs, which results in activation of the GTPase ROP11. ROP11, in turn, physically interacts with ABI2, a critical signal downstream of the ABA receptor, thereby suppressing the ABA response. Recent reports also revealed that FER interacts directly with both ABI1 and ABI2 (Chen et al., 2016), but until now, the biological function of the interaction between FER and ABI1 was unknown. In this study, we showed that FaMRLK47 interacts directly with FaABI1 (**Figure 7**). Moreover, we found that FaMRLK47 changes the sensitivity of ripening-related genes to ABA treatment (**Figure 6**). These results imply that FaMRLK47 suppresses ABA-induced gene expression by interacting with FaABI1. In *Arabidopsis*, FER has been shown to control pollen tube growth and fertilization (Huck et al., 2003). Future studies should examine whether FaMRLK47 also controls pollen tube growth and fertilization in strawberry.

Fruit quality is primarily determined by the composition of organic constituents, including sugars, organic acids, pigments, and volatile compounds. Fruit ripening is tightly associated with fruit quality formation. The present study not only shows that FaMRLK47 plays a crucial role in regulating fruit ripening progression, but also that it plays a role in modifying fruit quality, as evidenced by its function in regulating sugar (especially sucrose) metabolism. As shown in **Figure 5**, RNAi-mediated downregulation of *FaMRLK47* resulted in a large decrease in sucrose and fructose content, and overexpression of *FaMRLK47* appeared to increase the content of sucrose, fructose, glucose, and starch. In *Arabidopsis*, FER was reported to regulate starch content via a physical interaction with glyceraldehyde-3-phosphate dehydrogenase (Yang et al., 2015). A recent study in rice showed that DRUS1/2, the ortholog FERONIA in rice, influences sugar utilization or conversion (Pu et al., 2017). In the present study, we found that FaMRLK47 is an important regulator of sucrose and starch metabolism, indicating that FER-like protein kinases have somewhat similar roles in different species. Given that FaMRLK47 functions in sucrose metabolism and that FER functions in starch metabolism (Yang et al., 2015), the FER-like protein kinases appear to regulate sugar metabolism via different mechanisms. The mechanism by which FaMRLK47 regulates sucrose metabolism merits further investigation.

The involvement of FaMRLK47 in the regulation of strawberry fruit development and ripening indicates that FER-related protein kinases are versatile regulators of plant growth and development. Consistent with this, it has been suggested that *Arabidopsis* FER proteins are involved in a variety of important processes, such as root hair elongation (Duan et al., 2010; Huang et al., 2013), ethylene biosynthesis (Mao et al., 2015), starch accumulation (Yang et al., 2015), seed development (Yu et al., 2014), pathogen resistance (Keinath et al., 2010; Kessler et al., 2010), and vegetative growth (Guo et al., 2009a,b; Deslauriers and Larsen, 2010). A recent study showed that FER was transcriptionally downregulated by ethylene during post-harvest ripening and senescence of apple fruit (Zermiani et al., 2015), implying that FER also affects the ripening of climacteric fruits. Ethylene and ABA were demonstrated to be the main regulators of climacteric and non-climacteric fruit ripening, and the results of both the Zermiani study and our study suggest that FER functions in the cross-talk between ethylene and ABA. The diverse signaling mechanisms of FER in fruit ripening need to be further explored.

While the results of the present study suggest that FaMRLK47 is a critical regulator of strawberry fruit development and ripening, it should be noted that FaMRLK47 may regulate different biological processes, such as early fruit growth and development, onset of fruit ripening, and fruit quality formation. It will be of great significance to establish how these different biological processes are mediated by the same signal, FaMRLK47. ABA has been demonstrated to promote sugar accumulation in strawberry fruits (Jia et al., 2011). Given that overexpression of FaMRLK47 suppresses the ABA response, the upregulation of *FaSS* and *FaSPS1* and the increase in sucrose content observed in FaMRLK47 overexpression lines clearly do not occur via the ABA signaling pathway.

**FIGURE 8:**
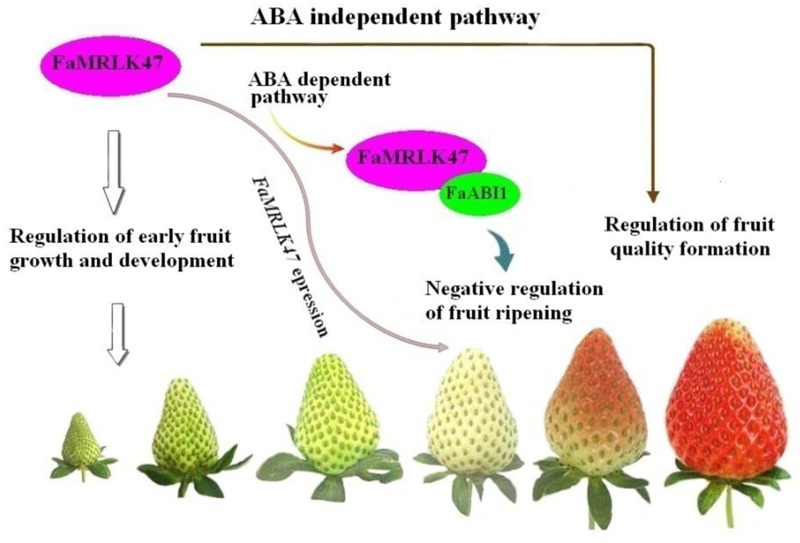
Proposed model for the role of FaMRLK47 in the regulation of strawberry fruit development and ripening. Acting as a negative signal, FaMRLK47 delays fruit ripening most probably by modifying the ABA signaling pathway. Furthermore, FaMRLK47 functions to regulate sugar metabolism, thereby regulating fruit quality formation in an ABA-independent pathway. Given the pivotal role of CrRLK1-related receptor kinases in the control of cell growth and the unique profile of FaMRLK47 expression during fruit development and ripening, FaMRLK47 is also expected to regulate early fruit growth and development.

In summary, the present study demonstrates that FaMRLK47 plays important roles not only in the regulation of strawberry ripening, but also in the regulation of fruit quality formation. Evidence for this is mainly derived from the following observations: (1) Overexpression and RNAi-mediated downregulation of *FaMRLK47* delayed and accelerated fruit ripening, respectively; (2) FaMRLK47 function is associated with ABA signaling, which is a major mechanism regulating strawberry fruit ripening. Specifically, FaMRLK47 physically interacts with ABI1, a key signal in the ABA signaling pathway, and manipulation of *FaMRLK47* expression modulated ABA-induced expression of ripening-related genes; (3) Manipulation of *FaMRLK47* expression modulated sucrose content and the expression of genes encoding key enzymes in sucrose metabolism. As sucrose metabolism affects strawberry fruit quality formation, FaMRLK47 is a regulator of strawberry fruit quality formation. We propose that FaMRLK47 influences fruit ripening and quality via two distinct pathways, the ABA-dependent pathway and ABA-independent pathway (**Figure 8**). Thus, this study provides insight into the molecular mechanisms underlying the regulation of strawberry fruit development and ripening.

## Author Contributions

MJ performed most of the experiments; ND, QZ, SX, and LW provided assistance with some of the experiments; YZ, PD, WM, and JL provided technical assistance; BL designed the experiments and analyzed the data; WJ conceived the project, supervised the experiments, and complemented the writing.

## Conflict of Interest Statement

The authors declare that the research was conducted in the absence of any commercial or financial relationships that could be construed as a potential conflict of interest.

## Footnotes

1 www.arabidopsis.org

2 https://www.ncbi.nlm.nih.gov/

3 http://www.primer3plus.com/cgi-bin/dev/primer3plus.cgi
