# Supplementary material for: A FERONIA-Like Receptor Kinase Regulates Strawberry (Fragaria × ananassa) Fruit Ripening and Quality Formation
Source: Front Plant Sci. 2017 Jun 28;8:1099. doi: 10.3389/fpls.2017.01099 (PMC5487432; doi:10.3389/fpls.2017.01099)
Supplement: Supplementary file 1 [file Data_Sheet_1.DOCX]

**Supplemental Table S1**: Primers used to amplify *FaMRLK47* and *FaMRLK50* and GenBank accession numbers.

| **Gene** | **Forward primer (5’-3’)** | **Reverse primer (5’-3’)** | **Genebank**  **accession number** | |
| --- | --- | --- | --- | --- |
| *FaMRLK47-*OE | TCTAGAATGAAGTGTTTCTTTTTCTATATTTG | GAGCTCTCAACGTCCCTTTGGGTTC | | KX374339 (XP_004302537.1) |
| *FaMRLK50-*OE | TCTAGAATGAAACTCATTCTCGCTCCTC | GAGCTCTCATCTCACAGTGGGATCCTTGATC | | KX374340 (XP_004306057.1) |
| *FaMRLK47-*RNAi-1 | CCATGGTTTCCGCAAATTACATCCCATCTG ATAA | GGCGCGCCGGGCTTAAACGTGACATCTAAAGACTCC | | KX374339 (XP_004302537.1) |
| *FaMRLK47-*RNAi-2 | GGATCCTTTCCGCAAATTACATCCCATCTGATAA | CCCGGGGGGCTTAAACGTGACATCTAAAGACTCC | | KX374339 (XP_004302537.1) |

**Supplemental Table S2**: qRT-PCR primers used to detect *FaMRLKs* expression.

| **Gene** | **Forward primer (5’-3’)** | **Reverse primer (5’-3’)** | **Genebank accession number** | |
| --- | --- | --- | --- | --- |
| *FaMRLK47* | GCTTAGGTCTTGGTATGATGA | TAGTTGAGGTTGATGTTGTTATTC | | KX374339 |
| *FaMRLK50* | TTGCCGTGAGTTGTATGAA | TTAGTCGTTCTGCTGTTCTT | | KX374340 |
| *FaMRLK53* | CGAAGAAGAAGCCGACATA | GCAATTACACCAGCAACAA | | KX374341 |
| *FaMRLK52* | AATCCAAGCATTCAACTCAAG | ACCACTCCATCCGACTAT | | KX374342 |
| *FaMRLK54* | AGCACAGAACGACACAAT | TCCACCACTCCAGAGAAT | | KX374343 |
| *FaMRLK51* | GCCATCTTGAACGGACTA | CTACACCTGCGATAATACCA | | KX374344 |
| *FaMRLK54* | TCACGCCATCATCAACTC | TGTCTGTATCAACCTCTTCATT | | KX374343 |

**Supplemental Table S3：**qRT-PCR primers used to detect the expression of ripening-related genes.

| **Gene** | **Full Name** | **Forward primer (5’-3’)** | **Reverse primer (5’-3’)** | |
| --- | --- | --- | --- | --- |
| *FaC4H* | cinnamate-4-hydroxylase | ACGCTCAACAGAAAGGAGAGAT | | TTCGGGGTGGTTCACAA |
| *Fa4CL1* | 4-coumarate:CoA ligase 1 | CGTAGACCCTGAAACTGGTG | | GTGTAGCCATCCTTCCTTGTC |
| *Fa4CL2* | 4-coumarate:CoA ligase 2 | ACGAATCCCTTCCCAGAAA | | TCTTTGTCTATGGTCCTCTCAGTC |
| *FaCHS* | chalcone synthase | CATACCCCGACTACTACTTTCGT | | CGCACATACTGGGATTCTCTT |
| *FaCHI* | chalcone isomerase | AGCGAAAGCCATTGAAAAGT | | CATTTGGTGATTGTGTGAAGAG |
| *FaF3H* | flavanone 3-hydroxylase | CTTTCGTGGTGAATCTTGGAG | | TCGCTATGGACAACCTGCT |
| *FaDFR* | dihydroflavonol 4-reductase | ACCCTGAGAACGAAGTGATAAAG | | TAAACACCACCCTCCGAACT |
| *FaUFGT* | flavonoid 3-O-glucosyltransferase | TAGAGGATGTGTGGAAGATTGGT | | CTGTTGTGCGAGTTGTTTTAGTG |
| *FaANS* | anthocyanidin synthase | CTTGGCTTGGGATTAGAAGAAG | | TGAGGGCATTTTGGGTAGTAGT |
| *FaPAL* | phenylalanine ammonia-lyase | CTTCTGTGGTGCTGTTTGATG | | AGGGTGGTGCTTCAGTTTATGT |
| *FaPE* | pentaerythritol | GGTTTCTACTGGTGCTGGTTTT | | CTCGGACTGTATCGTGTTGC |
| *FaPL* | pectate lyase | TCAACTCGTCAATGGCAGAC | | GAATGCTCGTATCAACCAGAGA |
| *FaPG* | polygalacturonase 1 | GCAAGTAGAGTCGCACAGTTTT | | TCAGTATTAGGCTTCCCACCA |
| *FaCEL* | carboxyl ester lipase | GCTCTGTTTTGCCTGGACTT | | GCGTGGCTTAGATAGTTGGAAT |
| *FaXYL1* | alpha-xylosidase 1 | ATGGAAAGCCTACTTGTGCTG | | CTGGTGTAATGTTGTTGGTCGT |
| *FaEXP1* | expansin 1 | AGGACGGAGTTGGATTGC | | TGAGCGTGAGCGTGAAG |
| *FaEXP2* | expansin 2 | GTATCGTCCCCGTCTCATTC | | AGTAGGAGTGCCCGTTGATT |
| *FaEXP3* | expansin 3 | TCACTGCCACTAACTTCTGC | | TTATGCCTCCTGCTCTCCT |
| *FaQR* | quinone oxidoreductase | CACTGACTCTCCCCTACCTACAAT | | ATACACTTCATCCCCCACCTTA |
| *FaSS1* | sucrose synthase | CCCTGATTCTGACCTTTACTGG | | GATGATGAAGTCGGTGTGGTT |
| *FaSPS1* | sucrose phosphate synthase 1 | CGTAGATTGGAGTTATGGAGAGC | | CGAATGATGTAAGAACCACTGC |
| *FaMYB10* | myb domain protein 10 | CAACAGCACCACCACAGACT | | GCTTGCCGATTGTACCGTAT |
| *FaNCED1* | 9-cis-epoxycarotenoid dioxygenase 1 | ACTGCTTCTGCTTCCATCTCT | | AGACACTCGTCGCATTCATT |
| *FaNCED2* | 9-cis-epoxycarotenoid dioxygenase 2 | GACACCTTTTGCTTCCACTTG | | GGATTTCGGACAACACACTCT |
| *FaNCED3* | 9-cis-epoxycarotenoid dioxygenase 3 | CAAACCCTATCACCCTTCTCAC | | TGGGCAACTTCTGCTTCTTT |
| *FaNCED4* | 9-cis-epoxycarotenoid dioxygenase 4 | GAACCGTGGCCCAAAGTTTC | | TCACGGCGTTCACAATCTGA |
| *FaAAO3* | aldehyde oxidase 3 | ACCGGATATCGTCCCATTGC | | CAAACACGACGAGGACCTGA |
| *FaZEP1* | zeaxanthin epoxidase 1 | ACTCCTCCTGACATTGCTGC | | TCTGAGAGCCTGCAACTTGG |
| *FaZEP2* | zeaxanthin epoxidase 2 | CCCGATTCCGATCACCAGAG | | CAAAGCGAAAACCAGCCCTC |
| *FaCYP707A1* | cytochrome P707A1 | CATATCAACGCCACAGCTGC | | ATGTTCCTCAGCCTCTCCCT |
| *FaCYP707A2* | cytochrome P707A2 | CCCTCCAGGCTCATTTGGTT | | GCTTTTGGGATAGGTGGGCT |
| *FaCYP707A3* | cytochrome P707A3 | CTGTTTGCAGCCCAAGACAC | | GCTGGCCATCCTCAAACTCT |
| *FaBG1* | beta glucosidase 1 | TGAGAATGGCATGGACGACC | | ATATGGAATTCCCGTCCGGC |
| *FaBG2* | beta glucosidase 2 | TGGATGGAGGGATGGTGGAT | | TGGCCATTCTTGCGTACAGT |
| *FaBG11* | beta glucosidase 11 | GCAAACCTACCAGCACAAGC | | ACTGGTGAACGTCGGAAGTC |
| *FaBG24* | beta glucosidase 24 | CAGGCAGGTGTTCAGTACGA | | CGATTTCGCTGCAGCTTTGT |
| *FaPYR1* | pyrabactin resistance 1 | GCTTCGTAATGAGCGTGGGA | | CTCTCCTCGTCCAGCAAGTC |
| *FaPYL2* | pyrabactin resistance 2 | CAAGAGCTGCAACATGAGCG | | ATCTCTAGCCTCTCGGTGCT |
| *FaPYL10* | pyrabactin resistance 10 | CGTTTGGTCAGTCATTCGCC | | ATGCTTCCTATGCCTCCGTC |
| *FaPYL12* | pyrabactin resistance 12 | CCGGGGAGTAGCAGTATGGA | | GCAAGCTTGTGATCACCACC |
| *FaABI1* | aba insensitive 1 | CAAGAGCCATTCTTTGTCGT | | TGGAATAATCCAGGGTTTCA |
| *FaABI2* | aba insensitive 2 | CGTATGCATTTGGCTCTGGC | | CCACGACACAGAACTGCTCT |
| *FaABI3* | aba insensitive 3 | CGGCGCCTGTATTAGTCCC | | TGCAGTCTCCAGCGTTTGAT |
| *FaABI4* | aba insensitive 4 | TCCTCATCACCACCGTCTT | | ACTCTGGCTCGTTTGCTCT |
| *FaABI5* | aba insensitive 5 | GGAGCTGGCAATGGTCG | | AGGCCCGCCTTTCCTT |
| *FaSnRK2.1* | SNF1-related protein kinase2.1 | CACAACCCAAATCAACTGTAG | | GGATAAGCACCAACCAGCAT |
| *FaSnRK2.2* | SNF1-related protein kinase2.2 | CCAGATAGTGTGCGAGTTTCAG | | ACTTCCTCCTTCCTTCATTTCC |
| *FaSnRK2.3* | SNF1-related protein kinase2.3 | TGATGACGGACAAGCAGAC | | TGCCTCAACGACCTATGATT |
| *FaSnRK2.4* | SNF1-related protein kinase2.4 | GCTGGAAAACACACTCTTGG | | CGGAGCAATGTAAGCAGGT |
| *FaSnRK2.5* | SNF1-related protein kinase2.5 | ATGAGATGAACAATCCAGTCCA | | TAGCACACAAAATCACCACTTG |
| *FaSnRK2.6* | SNF1-related protein kinase2.6 | GCTACACTCGCAACCAAAATC | | ACCCCACAAGACCAGACATC |
| *FaSnRK2.8* | SNF1-related protein kinase2.8 | GCATTCACGACCAAAGTCAA | | CAAGACCATACATCTGCCAACT |
| *FaSnRK2.9* | SNF1-related protein kinase2.9 | TACCCAAACACAGTTACGCATC | | GGCAACGAAAATCACCACTT |
| *FaMADS9* | MCM1,Agamous,Deficiens,and SRF box 9 | ACGCTGAGGTTGCTCTCATC | | AGGACTCTAGCTGACGCTCA |
| *FaACTIN* | actin protein | GCCAACCGTGAGAAGATG | | TCCAGAGTCAAGAACAATACCAG |

**Supplemental Table S4**: Primers used to amplify *FaMRLK47* and *FaABI1* in yeast two-hybrid assays.

| **Vector** | **Forward primer (5’-3’)** | **Reverse primer (5’-3’)** |
| --- | --- | --- |
| Y2H-GAD-FaABI1 | CATATGATGGAGGAGATGTCACC | GGATCCTCATGTTTTACTTTTAAACTTC |
| Y2H-GBD-FaMRLK47 | GGATCCAGAAGTTGCGCTTCCTCCCTCC | GAGCTCTCAACGTCCCTTTGGGTTCATGATTTG |

**Supplemental Table S5**: Primers used for BiFC and subcellular localization assays.

| **Gene** | **Forward primer (5’-3’)** | **Reverse primer (5’-3’)** |
| --- | --- | --- |
| pCambia::FaABI1-YFP^n^ | GGTACCATGGAGGAGATGTCACC | GGATCCTGTTTTACTTTTAAACTTCCTC |
| pCambia::FaMRLK47-YFP^c^ | GTCGACATGAAGTGTTTCTTTTTCTATATTTGGTTC | ACTAGTACGTCCCTTTGGGTTCATGATTTGTGAG |
| pMDC83::FaMRLK47-GFP | TTAATTAAATGAAGTGTTTCTTTTTCTATATTTGGTTC | GGCGCGCCAACGTCCCTTTGGGTTCATGATTTGTGAG |

**Supplemental Figure S1**


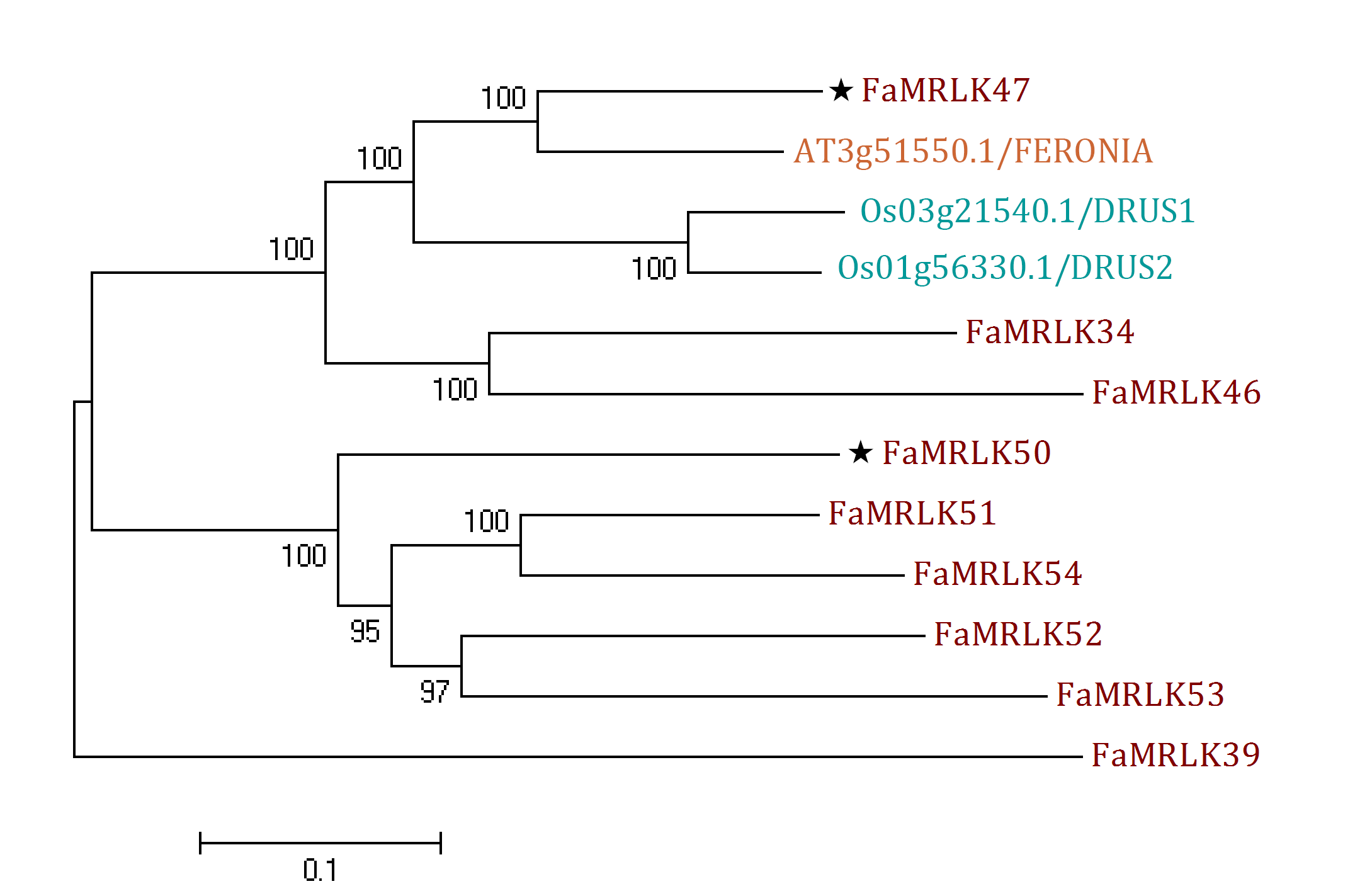


**Figure S1 |** **Rooted phylogenetic tree of FaMRLKs.** The phylogeny was constructed using the Neighbor-Joining method in MEGA4.0.2 with 1,000 bootstrap replicates. Fa, *Fragaria ananassa;* At, *Arabidopsis thaliana*; Os, *Oryza sativa.*
